# Examining the effect size and duration of retrieval-induced facilitation

**DOI:** 10.1007/s00426-022-01729-0

**Published:** 2022-08-30

**Authors:** Mercedes T. Oliva, Benjamin C. Storm

**Affiliations:** University of California, Santa Cruz, Santa Cruz, USA

## Abstract

Under certain conditions, the retrieval of some information can increase the recall of other information, a phenomenon known as retrieval-induced facilitation. Chan (Journal of Memory and Language 61:153–170, 2009) proposed two moderating factors to account for why retrieval causes facilitation in some situations and forgetting in others: (1) integration at the time of encoding and (2) the delay between retrieval practice and final test. Chan found a 9–11% facilitation effect when the materials were well integrated and the final test occurred after a 24-h delay. Two sets of experiments sought to replicate and extend Chan’s study by examining retrieval-induced facilitation not only following a 24-h delay but after longer delays (i.e., 1 or 2 weeks). A meta-analysis including these replications and the original experiments was also conducted. The results provide additional evidence of retrieval-induced facilitation, with no evidence that the effect varies as a function of the final delay. However, the size of the effect was found to be somewhat smaller than previously observed.

## Introduction

Tests have been documented to improve the later recall of tested information (i.e., the testing effect), making them a learning tool that can be implemented easily and effectively (Roediger & Karpicke, 2006a). Tests allow learners to practice retrieving information in a way that potentiates the subsequent retrieval of that information. However, it is often implausible (if not impossible) to create a practice test that includes all to-be-learned information, necessarily leaving some subset untested. The question of what happens to untested information has been studied extensively over the years, and many results point to a phenomenon known as retrieval-induced forgetting (Anderson et al., 1994).

In the paradigm typically used to study retrieval-induced forgetting (Anderson et al., 1994), participants are asked to memorize a list of category–exemplar pairs and are then asked to retrieve from memory half of the exemplars from half of the categories. This selective retrieval practice creates three types of items: (1) practiced items, Rp+, (2) unpracticed items from practiced categories, Rp−, and (3) unpracticed items from unpracticed categories, Nrp. After a brief delay, participants are tested on all the items. Due to the testing effect, Rp+ items are typically recalled best. For current purposes, however, the more important finding is that Rp− items are typically remembered worse than Nrp items (i.e., retrieval-induced forgetting). According to the inhibitory account of retrieval-induced forgetting (Anderson, 2003; Storm & Levy, 2012), Rp− items are inhibited during retrieval practice to facilitate access to Rp+ items, with this inhibitory effect persisting until the final test when the Rp- items become the targets of recall themselves. Retrieval-induced forgetting is argued to be an adaptive phenomenon in memory as it allows people to access relevant information more effectively by minimizing the likelihood that other, less relevant information will interfere during retrieval. Other theoretical accounts of retrieval-induced forgetting have argued that retrieval causes forgetting through non-inhibitory mechanisms, such as strength-based interference, blocking, or changes in context that make it difficult to retrieve the Rp− items (Jonker et al., 2013; Raaijmakers & Jakab, 2013; Verde, 2013).

Importantly, not all research has observed evidence of retrieval-induced forgetting. Under certain conditions, retrieval practice has been shown to enhance the subsequent remembering of related non-practiced information, a phenomenon referred to as retrieval-induced facilitation. For example, Chan et al. (2006) asked participants to study information about a particular topic (e.g., the toucan). Some participants received retrieval practice for the information, whereas others did not. After a 24-h delay, participants who received retrieval practice performed better on a final test not only for the information that was practiced, but also for related information that was not practiced. Although seemingly at odds with evidence of retrieval-induced forgetting, demonstrations of retrieval-induced facilitation fit well with the idea that retrieval should benefit memory for related non-retrieved information via a kind of spreading activation (Anderson, 1983; Collins & Loftus, 1975). Specifically, when two elements in memory are related and well integrated, the activation of one element should be expected to activate the other element, making it more recallable (not less recallable) in the future than it would have been otherwise.

Why do some studies using a retrieval-practice paradigm show evidence of retrieval-induced facilitation and not retrieval-induced forgetting? Chan et al.’s (2006) experiments differed in two critical ways from many studies showing retrieval-induced forgetting. First, the authors designed the study materials to reflect educational materials. As such, the materials were well integrated, meaning that the content and presentation style were cohesive and clear, and participants were encouraged to consider the many pieces of information in relation to one another. This format is a departure from the more typical retrieval-practice paradigm (Anderson et al., 1994; Murayama et al., 2014), which tends to use category–exemplar pairs designed to be less integrated. The integrated materials and the instructions directing participants to connect the various pieces of information may work together to prompt participants to encode the material in a way that serves as a boundary condition on retrieval-induced forgetting (Anderson, 2003; Storm et al., 2015). Indeed, there is evidence that retrieval-induced forgetting is not observed in the typical paradigm when Rp+ and Rp− items are well integrated, either because of how the materials are constructed or because of the way participants are instructed to encode them (e.g., Anderson & McCulloch, 1999; Anderson et al., 2000; Goodmon & Anderson, 2011).

The second key feature of the experiments reported by Chan et al. (2006) was the length of the delay between retrieval practice and final test. Their study used a 24-h delay, which has been argued to be enough time to allow the effects of retrieval-induced forgetting to dissipate (e.g., Carroll et al., 2007; MacLeod & McCrae, 2001; Saunders et al., 2009; Saunders & MacLeod, 2002). Although more recent work has suggested that retrieval-induced forgetting can, in some situations, persist as long as a week following retrieval practice (García-Bajos et al., 2009; Migueles & García-Bajos, 2007; Saunders et al., 2009; Storm et al., 2012), it stands to reason that the inhibitory effects of retrieval practice should, at the very least, become less pronounced after a long delay than after a short delay. This should make it less likely for retrieval-induced forgetting to offset or mask any co-occurring effect of retrieval-induced facilitation (which is to say that there is no theoretical reason to think that only one of these effects can be present at a given time, but rather that they cannot be observed simultaneously). Beyond that, a delay may enhance the effect of retrieval-induced facilitation in the same way that it has been shown to enhance the testing effect more generally. Specifically, when Rp− items are co-activated during retrieval practice (owing to their relatedness or high level of integration), they may benefit from a type of covert testing effect that is more likely to enhance recall after a long delay than after a short delay (e.g., Congleton & Rajaram, 2012; Roediger & Karpicke, 2006b).

In summary, Chan et al. (2006) simultaneously implemented two distinct conditions—high integration and a long delay—the combination of which may have diminished retrieval-induced forgetting and made it more likely for retrieval-induced facilitation to be observed. In a subsequent study, Chan (2009) attempted to isolate the effects of integration and delay by manipulating them experimentally. In the first of two experiments, Chan employed the same materials as were used by Chan et al. using a 2 (Integration: low, high; between subjects) × 2 (Delay: 20-min, 24-h; between subjects) × 3 (Item Type: Rp+, Rp−, Nrp; within-subjects) mixed design. The to-be-learned prose passages were either presented as they would typically be structured (high-integration condition) or with the sentences within each paragraph shown in a random order (low-integration condition). In the high-integration condition, participants also received instructions to integrate the information as it was presented. In the low-integration condition, the materials were presented as a collection of facts, and participants were not instructed to integrate the information. Consistent with prior research on retrieval-induced forgetting, participants in the low-integration/20-min condition demonstrated significant retrieval-induced forgetting (*M* effect size = − 9%). In comparison, participants in the high-integration/24-h condition (the same condition used in the 2006 experiments) demonstrated significant retrieval-induced facilitation (*M* effect size = 9%). Interestingly, the participants in the other two conditions (high-integration/20-min and low-integration/24-h) did not show either effect to a level of statistical significance, indicating that both high integration and a long delay may be required to observe retrieval-induced facilitation.

Chan’s (2009) Experiment 2 sought to investigate the same factors with a different type of materials. Rather than prose passages, object–location pairs that formed to prepositional sentences were used. For example, for the object–location pair “orange–fridge”, participants would be presented with *“The orange is in the fridge.”* Integration was manipulated by either presenting objects sequentially in the same location (i.e., blocked by location) and giving the high-integration instructions (high-integration condition), or by presenting the sentences in a random order and not giving the high-integration instructions (low-integration condition). A final test took place following a 20-min or 24-h delay. Participants showed the same pattern as in Experiment 1: participants in the low-integration/20-min condition showed retrieval-induced forgetting (*M* effect size = − 7%). Participants in the high-integration/24-h condition showed retrieval-induced facilitation (*M* effect size = 11%). Participants in the other two conditions did not show evidence of either effect.

Despite the applied and theoretical importance of Chan’s (2009) findings, we are not aware of any published attempts to directly replicate them. Although there is strong evidence of integration and delay acting as boundary conditions to prevent or diminish the effect of retrieval-induced forgetting, there is much less evidence of the two factors combining to produce retrieval-induced facilitation. Moreover, the original experiments reported by Chan were somewhat underpowered, making it even more important to investigate the finding’s reliability and to estimate the effect size more accurately. As such, the first goal of the current study was to perform a partial direct replication of Chan’s high-integration/long-delay conditions. Specifically, the current study focused on the conditions under which Chan observed retrieval-induced facilitation. The other conditions were not included, as evidence of retrieval-induced forgetting is already well established, as well as the mitigating effects of integration and delay (Murayama et al., 2014; Storm et al., 2015).

A second goal of the current study was to examine the durability of the retrieval-induced facilitation effect, replicating not only the 24-h delay condition used by Chan (2009) but also implementing longer delays (i.e., 1 or 2 weeks). Together, these conditions provide opportunities to replicate and extend Chan’s findings, and to determine whether the relatively large effect of retrieval-induced facilitation (9–11%) which was documented after a 24-h delay would persist 1–2 weeks following retrieval practice. Achieving a better understanding of how long retrieval-induced facilitation persists will shine a light on the everyday implications of selective retrieval practice and provide new insight into the effect’s theoretical mechanisms.

On the one hand, the retrieval-induced facilitation effect may exhibit a pattern such that it is strongest after a 24-h delay but decreases steadily after that. From a theoretical standpoint, however, this possibility seems unlikely. Retrieval-induced facilitation may be long-lasting and perhaps persist indefinitely. Whereas retrieval-induced forgetting is considered the result of a temporary reduction in accessibility, retrieval-induced facilitation is believed to reflect the consequences of unpracticed items being activated by retrieval, thus benefiting from a type of retrieval practice effect. Therefore, just as the testing effect has been shown to persist (and even become larger) following long delays (Butler & Roediger, 2007; Roediger & Karpicke, 2006b; Wheeler et al., 2003), retrieval-induced facilitation may be expected to follow a similar trajectory. That said, with delay, overall recall rates begin to decline, which may make it more challenging to observe a large effect after a long delay than after a short delay. At a minimum, however, based on the theoretical explanation of retrieval-induced facilitation provided by Chan (2009), the effect should remain significant even after a longer final delay.

## Experiments 1a and 1b

The goal of the first set of experiments was to replicate and extend the original findings of Chan (2009). Every participant took part in two separate experiments (Experiment 1a and Experiment 1b), run sequentially within a single 1-h time block. Experiment 1a was designed to replicate the high-integration/long-delay condition of Chan’s Experiment 1, with participants studying and receiving retrieval practice for a subset of items from one of two prose passages. Experiment 1b was designed to replicate the high-integration/long-delay condition of Chan’s Experiment 2, with participants studying and receiving retrieval practice for a subset of object–location pairs. Half of the participants were tested on both sets of materials after a 24-h delay, thus replicating the delay used by Chan. The other half of the participants were tested after a 2-week delay. Retrieval-induced facilitation was assessed by comparing the final test performance on unpracticed items from practiced passages/locations (Rp− items) to that of unpracticed items from unpracticed passages/locations (Nrp items).

### Method

#### Participants

Seventy-four University of California, Santa Cruz (UCSC) undergraduate students received partial course credit in a psychology course for their participation. Participants were assigned pseudo-randomly to participate in either the 1-day Delay condition or the 2-week Delay condition. Specifically, assignment to Delay condition was determined based on each participant’s response to an arbitrary question on an online pre-screening survey completed at the beginning of the term. The question asked students to indicate whether the number on their clock was odd (e.g., 2:31 pm) or even (e.g., 2:32 pm) when they were answering the question. Students who indicated an odd number were invited to sign up for the 1-day condition. Students who indicated an even number were invited to sign up for the 2-week condition. Participants were only aware of the condition to which they were assigned and, thus, only had the opportunity to sign up for that condition.

Ten participants failed to return for the second part of the study (three participants in the 1-day condition; seven participants in the 2-week condition). These participants were not included in the analysis, leaving a total of 33 and 31 participants in the 1-day and 2-week conditions, respectively. The stopping rule, which was determined before commencing data collection, was to stop when we reached a total of 64 participants. A power analysis confirmed that this total number would be sufficient for the study to have approximately 80% power to detect a 6% effect size in the overall main effect of retrieval-induced facilitation in each of the two sub-experiments, assuming a standard deviation of the effect size to be 17%. The number of participants in the 1-day condition was also expected to be sufficient to have 80% power to detect the 9% effect size initially observed by Chan (2009) in that condition alone. For comparison purposes, Chan’s study included a sample of 24 participants in each of the high-integration/long-delay conditions reported in the two experiments.

#### Design

The experiments were run in-person, either individually or in pairs, in a contained room with a barrier between two side-by-side computers. A research assistant was always available to answer questions and monitor and facilitate progress.

In Session 1, participants completed the study and retrieval-practice phases for Experiments 1a and 1b. In Session 2, which took place after a one-day or two-week delay, they completed the final tests for both Experiments 1a and 1b. Both Sessions took place in the same room under similar circumstances (testing was completed alone or in pairs with a research assistant on hand). Participants run in pairs were always assigned to the same Delay condition.

All materials, including verbal instructions given to participants, were constructed to follow as closely as possible the instructions used in Experiments 1 and 2 of Chan (2009), based on the materials used in Experiments 2 and 3 of Chan et al. (2006).

Experiments 1a and 1b each consisted of 2 (Delay: 1 day, 2 weeks; between subjects) × 3 (Item Type: Rp+, Rp−, Nrp; within-subjects) mixed designs. Following the common practice of the retrieval-induced forgetting/facilitation literature: *Rp*+ items consisted of items that received retrieval practice; *Rp−* items consisted of unpracticed items from the practiced categories; *Nrp* items consisted of all items from the unpracticed categories.

#### Experiment 1a

##### Materials

*Prose passages*: Two passages were used (received through direct communication from Dr. Chan), one on the Big Bang Theory and one on the Shaolin Temple. Both passages were approximately 1900 words and 13 paragraphs. Like Chan (2009), the passages were divided into sentences, and individual sentences were inserted into Google Slides presentations such that each slide contained one sentence. Sentences were restructured from their original content so that they could stand on their own when presented individually (for example, changing “He” to “Arthur Eddington”).

*Retrieval practice questions*: The questions used for the retrieval practice stage of Experiment 1a were drawn directly from Chan (2009, Appendix C). Twenty-four questions (short-answer, often fill-in-the-blank) were created from each passage, consisting of 12 related pairs. An example of a related pair: *According to the flat and open models, the universe will…* (answer: expand infinitely) paired with *According to the oscillating closed universe model, the universe will alternate between a big bang and a…* (answer: big crunch). Four subsets of items were created to counterbalance (a) which passage received retrieval practice, and (b) which item from each related pair would be used for retrieval practice.

*Test questions*: The test consisted of all 24 possible questions for each passage. Test items were divided into three categories: (1) 12 items that received retrieval practice, Rp+; (2) 12 items that did not receive retrieval practice but were from the passage that received retrieval practice, Rp−; and (3) 24 items from the passage that did not receive retrieval practice, Nrp. The order of questions was counterbalanced between participants such that half of the participants began the test with the Rp+/Rp− questions, and the other half began with the Nrp questions.

*Procedure*: The procedure was the same as that of Chan (2009). Session 1 began with participants instructed to read the two passages for 16 min each (Big Bang Theory first, followed by Shaolin Temple) and it was explicitly noted that they should attempt to integrate the sentences as well as possible (Chan, 2009, Appendix E). They were also encouraged to read for the entire 16 min, starting again from the beginning if they reached the end before time elapsed. A research assistant informed participants when the time was up. Half of the participants completed retrieval practice for the Big Bang Theory, and the other half completed retrieval practice for the Shaolin Temple. Retrieval practice was completed immediately after reading the relevant passage.

For retrieval practice, a research assistant directed the participant to follow a URL to a Qualtrics survey with 12 questions (repeated twice) about the passage. In total, retrieval practice took 10 min. Participants had 25 s to answer each question before the computer automatically advanced to the next item. Following retrieval practice, participants were dismissed.

After their assigned delay, participants returned to the lab to complete the final test. Specifically, they were asked to answer 24 questions on the Big Bang Theory passage in the same format used for retrieval practice. The same process was repeated for the Shaolin Temple. The two tests took approximately 16 min.

#### Experiment 1b

##### Materials

*Object–location pairs*: Twenty-four object–location pairs were presented to participants individually in cohesive sentences (e.g., The rose is in the garden.) on a computer screen using PowerPoint. The object–location pairs consisted of six individual locations (e.g., garden), and each location had four individual objects (e.g., rose) reported to be in that location. The object–location pairs were generated randomly (MATLAB 2019b) for each participant, selected from a list of 24 possible objects and 24 possible locations, drawn from Chan (2009, Appendix D). No two objects beginning with the same two letters were placed in the same location (e.g., bottle and book were never both in the nursery).

*Sentences for retrieval practice*: In retrieval practice, participants saw a Qualtrics survey showing a subset of 8 of the object–location pairs with all but the first two letters of the object removed from the sentence (e.g., The ro_____ is in the garden.). These were a subset of object–location pairs that were randomly generated (MATLAB 2019b) to include half of the objects (2 of 4) from 4 (of 6) locations. Participants completed retrieval practice twice.

*Sentences for test*: For the test, participants saw a Qualtrics survey consisting of all 24 object–location pairs in the same style used for retrieval practice. The test items consisted of three item types: (a) the 8 object–location pairs that received retrieval practice (2 objects from 4 locations), Rp+; (b) the 8 object–location pairs that did not receive retrieval practice but were from the same locations as those that did receive retrieval practice, Rp−; and (c) the 8 object–location pairs from the completely unpracticed locations, 4 objects from 2 locations, Nrp.

*Procedure*: To begin the study phase of Experiment 1b, a research assistant opened the object–location pairs file while explaining the instructions, emphasizing the need to try to integrate the information as well as possible (Chan, 2009, Appendix E). All items in each location were presented successively before moving to the next location, increasing the ability of participants to integrate the object–location pairs. Specifically, the first four sentences described four objects in the same location; then, the following four sentences described four objects in another location, and so on. The study phase took less than 4 min (8 s per object–location pair, with 1 s between slides).

Participants completed the retrieval practice phase immediately after the study phase. Eight of the twenty-four object–location pairs were practiced (repeated twice, individually presented, and in random order) by typing the correct object name into a textbox. Retrieval practice took less than 3 min (10 s per item). After completing retrieval practice, participants were done for the day.

Participants were tested on the object–location pairs in Session 2, after their assigned delay. All 24 object–location pairs were presented in a random order using the same format used for retrieval practice, but with 15 s per item. The test took approximately 6 min.

### Results

#### Experiment 1a

An initial 3 (Item Type: Rp+, Rp−, Nrp; within-subjects) × 2 (Delay: 1 day, 2 weeks; between-subjects) mixed-design ANOVA was performed on the proportion of items successfully recalled on the final test. A significant effect of Item Type was observed, *F*(2, 124) = 32.04, *p* < 0.001, *η*_*p*_^2^ = 0.34. Pairwise comparisons revealed that Rp + items (*M* = 0.38, SE = 0.03) were recalled significantly better than both Rp− items (*M* = 0.22, SE = 0.02; *p* < 0.001, 95% CI_difference_ = [0.12, 0.21]) and Nrp items (*M* = 0.19, SE = 0.02; *p* < 0.001, 95% CI_difference_ = [0.13, 0.25]). The difference between Rp- items and Nrp items, however, was not significant (*p* = 0.34, 95% CI_difference_ = [− 0.02, 0.07]). The main effect of Delay was also significant, *F*(1, 62) = 18.31, *p* < 0.001, *η*_*p*_^2^ = 0.23, with performance in the 1-day Delay condition (*M* = 0.33, *SE* = 0.02) being better than for the 2-week Delay condition (*M* = 0.20, *SE* = 0.02, 95% CI_difference_ = [0.07, 0.20]). The interaction between Delay and Item Type was not significant, *F*(2, 124) = 0.42, *p* = 0.66, *η*_*p*_^2^ = 0.01.

As planned, and to test specifically for the presence of retrieval-induced facilitation, a 2 (Item Type: Rp−, Nrp; within-subjects) × 2 (Delay: 1 day, 2 weeks; between-subjects) ANOVA was run with only Rp− and Nrp items included in Item Type (omitting Rp+ items). There was no effect of Item Type, *F*(1, 62) = 0.93, *p* = 0.34, η_p_^2^ = 0.02, 95% CI_difference_ = [− 0.02, 0.07], indicating that there was no evidence for a main effect of retrieval-induced facilitation, but once again a main effect of Delay was significant, *F*(1, 62) = 26.41, *p* < 0.001, *η*_*p*_^2^ = 0.30, with participants in the 1-day delay condition (*M* = 0.28, *SE* = 0.02) performing significantly better than participants in the 2-week delay condition (*M* = 0.13, *SE* = 0.02, 95% CI_difference_ = [0.09, 0.20]). This analysis did not reveal a significant interaction, *F*(1, 62) = 0.72, *p* = 0.40,* η *_p_^2^= 0.01.

**Fig. 1:**
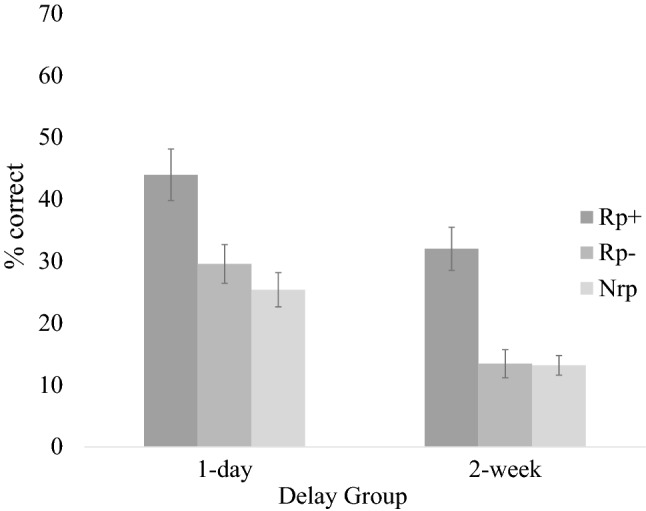
Experiment 1a test performance by delay

Separate ANOVAs compared the recall of Rp- and Nrp items for the two Delay conditions (See Fig. 1). Neither analysis showed significant evidence of retrieval-induced facilitation. The first ANOVA, for the 1-day Delay condition, *F*(1, 32) = 1.53, *p* = 0.23, *η*_*p*_^2^ = 0.05, 95% CI_difference_ = [− 0.03, 0.11], showed performance on Rp− items (*M* = 0.30, *SE* = 0.03) to be non-significantly higher than performance on Nrp items (*M* = 0.25, *SE* = 0.03). The second ANOVA, for the 2-week Delay condition, revealed performance on Rp− items (*M* = 0.13, *SE* = 0.02) to be nearly identical to performance on Nrp items (*M* = 0.13, *SE* = 0.02), *F*(1, 30) = 0.01, *p* = 0.93, *η*_*p*_^2^ = 0.00, 95% CI_difference_ = [− 0.06, 0.07].

#### Experiment 1b

Experiment 1b used the same analysis plan as Experiment 1a and showed a similar pattern of results.

**Fig. 2:**
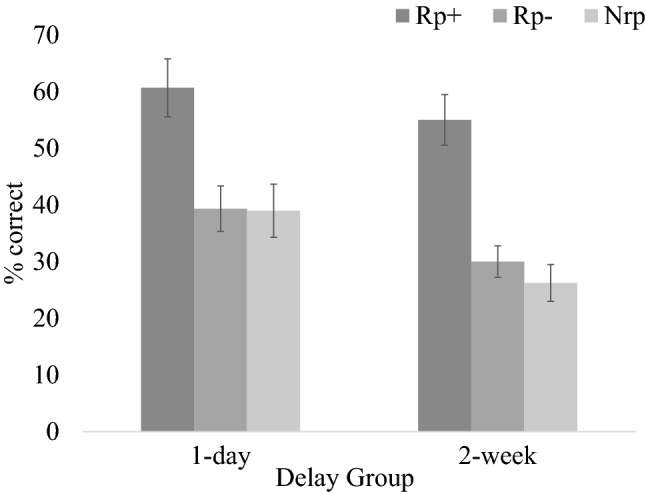
Experiment 1b test performance by delay. Error bars show standard errors

An initial 3 (Item Type: Rp+, Rp−, Nrp) × 2 (Delay: 1 day, 2 weeks) mixed-design ANOVA was performed on the proportion of items successfully recalled on the final test (Fig. 2). A significant main effect of Item Type was observed, *F(*2, 124) = 45.85,* p* < 0.001, *η*_*p*_^2^ = 0.43, with Rp+ items (*M* = 0.58, *SE* = 0.03) being recalled significantly better than both Rp− items (*M* = 0.35, *SE* = 0.03;* p* < 0.001, 95% CI_difference_ = [0.18, 0.29]) and Nrp items (*M* = 0.33, *SE* = 0.03; *p* < 0.001, 95% CI_difference_ = [0.19, 0.32]). The difference between Rp- items and Nrp items, however, was not significant (*p* = 0.48, 95% CI_difference_ = [− 0.04, 0.08]). The main effect of Delay approached significance, *F*(1, 62) = 3.53, *p* = 0.07, *η*_*p*_^2^ = 0.05, with participants in the 1-day Delay condition (*M* = 0.46, *SE* = 0.03) performing numerically better than participants in the 2-week Delay condition (*M* = 0.37,* SE* = 0.04). The interaction between Delay and Item Type was not significant: *F*(2, 124) = 0.73, *p* = 0.49, *η*_*p*_^2^ = 0.01.

To test specifically for the presence of retrieval-induced facilitation, the same ANOVA including only Rp− and Nrp items (omitting Rp+ items) was run. There was no main effect of Item Type, *F*(1, 62) = 0.50, *p* = 0.48, *η*_*p*_^2^ = 0.01, revealing no significant evidence of a difference in performance between Rp− items (*M* = 0.35, *SE* = 0.03) and Nrp items (*M* = 0.33, *SE* = 0.03), which is to say that there was no evidence of retrieval-induced facilitation. A main effect of Delay was present: *F*(1, 62) = 5.72, *p* = 0.02, *η*_*p*_^2^ = 0.08. Participants in the 1-day Delay condition (*M* = 0.39, *SE* = 0.03) performed significantly better than participants in the 2-week Delay condition (*M* = 0.28, *SE* = 0.03). The analysis did not reveal a significant interaction, *F*(1, 62) = 0.34, *p* = 0.56, *η*_*p*_^2^ = 0.01.

Separate ANOVAs tested for retrieval-induced facilitation by comparing the recall of Rp- and Nrp items in the two Delay conditions. Neither analysis showed evidence of the effect. For the 1-day Delay condition, *F*(1, 33) = 0.01, *p* = 0.93, *η*_*p*_^2^ = 0.00, 95% CI_difference_ = [− 0.08, 0.09], performance on Rp− items (*M* = 0.39, *SE* = 0.04) was almost identical to performance on Nrp items (*M* = 0.39, *SE* = 0.05). For the 2-week Delay condition, *F*(1, 29) = 0.91, *p* = 0.35, *η*_*p*_^2^ = 0.03), 95% CI_difference_ = [− 0.04, 0.12], performance on Rp- items (*M* = 0.30, *SE* = 0.03) was numerically, but not significantly, higher than performance on Nrp items (*M* = 0.26, *SE* = 0.03).

### Discussion

Although the results of Experiments 1a and 1b failed to provide significant evidence of retrieval-induced facilitation, both experiments showed some limited numerical evidence of the effect. Moreover, it is noteworthy that neither of the two experiments, which consisted of two types of materials (prose and object–location pairs) tested after two different delays (one day and two weeks), showed any evidence of retrieval-induced forgetting. This finding suggests that, at least under these testing conditions, the simultaneous implementation of high integration at the time of encoding and long delay between retrieval practice and test may be an effective tool to prevent retrieval-induced forgetting.

## Experiments 2a and 2b

Although a significant effect of retrieval-induced facilitation was not observed in Experiments 1a and 1b, the small numerical effect (and general directional consistency across conditions) lends itself to the possibility that an effect exists, but that is much smaller than that observed by Chan (2009). To provide further clarification, Experiments 1a and 1b were replicated with the addition of a new manipulation that could potentially make the study more sensitive to the benefits of retrieval practice on unpracticed items. Specifically, participants received feedback on the final test, thus giving them an additional opportunity to study the items before giving them a second final test moments later. Presumably, although Rp- items might not be more recallable than Nrp items on the first final test, the Rp- items might be better learned or have higher storage strength than Nrp items due to their previous activation (Bjork & Bjork, 1992), thus potentiating their relearning when the correct answers are provided via feedback. Said differently, if retrieval practice activates related items in memory, then it should act to not only potentiate the future accessibility/retrieval strength of related non-target items in memory (as evidenced by observations of retrieval-induced facilitation), but it should also act to potentiate the availability/storage strength of related non-target items in memory. Although this activation might not be sufficient to make Rp− items measurably more recallable than Nrp items, especially after a long delay, it could be sufficient to put Rp− items in a position to be more effectively relearned than Nrp items. Indeed, as predicted by the New Theory of Disuse (Bjork & Bjork, 1992), items with higher storage strength are expected to benefit more from relearning than items with lower storage strength (assuming equivalent levels of retrieval strength). Thus, even if Rp− items are not recalled better than Nrp items on an initial final test, when that test is followed by feedback in which the correct items are relearned, Rp− items might become better recalled than Nrp items on a second final test.

Experiments 2a and 2b replicated Experiments 1a and 1b with the addition of a second test following the first test (with the first test including feedback in the form of the correct answers). The first test served as a close replication of the earlier experiments, with the only difference being that participants were given feedback after attempting to recall each item. The second test was added to provide what theoretically could be a more sensitive measure of retrieval-induced facilitation. Another critical difference in Experiment 2 was the number of Delay conditions and how participants were assigned to them. In Experiments 2a and 2b, participants were randomly assigned to one of three Delay conditions (1 day, 1 week, 2 weeks), which allowed the possibility of a non-linear relationship between delay and retrieval-induced facilitation to be explored.

### Method

#### Participants

A total of 103 UCSC undergraduate students were recruited to participate in the study and received partial course credit for their participation. Unlike in Experiment 1, participants in Experiment 2 were randomly assigned to Delay condition. Specifically, participants were informed when they signed up to participate in the study that they would be randomly assigned to one of three conditions (1 day, 1 week, or 2 weeks). They were not informed of the final delay to which they were assigned until after completing the first session of the experiment, and none of the participants dropped out of the study when they were informed of the final delay. That said, not all participants completed the second session. All participants returned in the 1-day condition, but one participant failed to return in the 1-week condition, and three participants failed to return in the 2-week condition. These participants were not included in the analysis, leaving a total of 35, 31, and 33 participants in the 1-day, 1-week, and 2-week conditions, respectively. The stopping rule, determined before commencing data collection, was to stop at 96 participants, matching the number of participants per condition in Experiment 1 (three additional participants were run by accident, and we chose to include them in the data set before analyzing the data). A power analysis confirmed that this total number would be sufficient for the study to have 80% power to detect a 5.5% effect size in the overall main effect of retrieval-induced facilitation in each of the two sub-experiments, assuming a standard deviation of the effect size to be 19%.

#### Design

Participants took part in the experiment remotely. They completed the study and retrieval practice phases for both Experiments 2a and 2b in Session 1; they completed the final test for both experiments in Session 2, after either a 1-day, 1-week, or 2-week Delay. Links and instructions were emailed to the participants on the morning of their scheduled participation days.

The materials and procedure were identical to those described in Experiments 1a and 1b, except for minor alterations necessary to add the second final test and to allow the entire experiment (Session 1 and Session 2) to be completed remotely. Changes included (1) collapsing multiple surveys (with multiple links) into one link to ensure that participants completed the tasks in the correct order, (2) adding feedback to the first final test, and (3) adding a second final test (without feedback) after the first test. Both final tests took place in Session 2 and followed the same general method described in Experiments 1a and 1b. In the first final test, participants were provided the correct answer after every question. After completing the first final test, participants were given a filled 15-min distractor task (word search) before proceeding to the second final test.

The experiment employed a 3 (Delay: 1 day, 1 week, 2 weeks; between-subjects) × 2 (Item Type: Rp-, Nrp; within-subjects) mixed design. Participants were randomly assigned to a delay of either one day, one week, or two weeks between the date of encoding/retrieval practice (Session 1) and the date of the final tests (Session 2). For this experiment, Rp + items were not included in either test as they are not necessary for calculating retrieval-induced facilitation, and because of the possibility that testing Rp+ items on the first final test could disparately affect the recall of Rp- and Nrp items on the second final test.

### Results

#### Experiment 2a

**Fig. 3:**
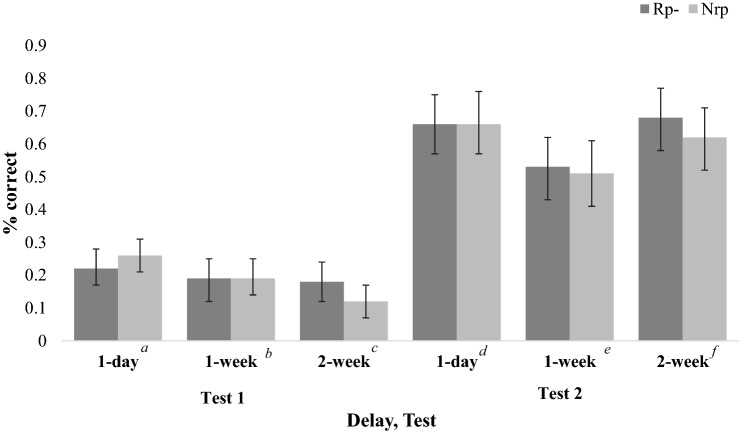
Experiment 2a performance by delay and test. Error bars show standard errors. ^a^*t*(34) = − 1.33, *p* = 0.19, 95% CI_difference_ = [− 0.09, 0.02], BF_01_ = 2.43. ^b^*t*(30) = − 0.21, *p* = 0.84, 95% CI_difference_ = [− 0.09, 0.07], BF_01_ = 5.12. ^c^*t*(32) = 2.33, *p* = 0.03, 95% CI_difference_ = [0.01, 0.11], BF_10_ = 1.91. ^d^*t*(34) = − 0.11, *p* = 0.92, 95% CI_difference_ = [− 0.07, 0.06], BF_01_ = 5.48. ^e^*t*(30) = 0.33, *p* = 0.75, 95% CI_difference_ = [− 0.09, 0.07], BF_01_ = 4.96. ^f^*t*(32) = 1.53, *p* = 0.14, 95% CI_difference_ = [− 0.02, 0.14], BF_01_ = 2.00.

A 2 (Item Type: Rp−, Nrp; within-subjects) × 2 (Test: Test 1, Test 2; within-subjects) × 3 (Delay: 1 day, 1 week, 2 weeks; between-subjects) ANOVA was performed. Only one main effect met the level of statistical significance. Specifically, for Test, performance was better on Test 2 (*M* = 0.61, *SE *= 0.03) than on Test 1 (*M* = 0.19, *SE* = 0.01; *F*(1, 96) = 420.63, *η*_*p*_^2^ = 0.81, *p* < 0.001, 95% CI_difference_ = [0.38, 0.46]). Delay did not meet the level of statistical significance, *F*(2, 96) = 2.73, *p* = 0.07, *η*_*p*_^2^ = 0.05, although numerically, participants in the 1-day Delay condition (M = 0.45, SE = 0.03) performed best, followed by participants in the 2-week (M = 0.40, *SE* = 0.03), and 1-week (M = 0.35, *SE* = 0.03) Delay conditions. Item Type was also not significant, *F*(1, 96) = 0.74, *p* = 0.39, *η*_*p*_^2^ = 0.01, 95% CI_difference_ = [− 0.02, 0.05], meaning that Rp− items were not recalled significantly better or worse than Nrp items. That is, there was no evidence of retrieval-induced facilitation (or forgetting) (Fig. 3)

There was a significant interaction between Test and Delay: *F*(2, 96) = 5.71, *p* = 0.01, *η*_*p*_^2^ = 0.11. Although all Delay conditions increased in performance from Test 1 to Test 2, the extent to which improvement occurred did vary between conditions. Performance for the 1-day Delay condition improved from 0.24 (*SE* = 0.02, 95% CI = [0.20, 0.29]) to 0.66 (*SE* = 0.04, 95% CI = [0.58, 0.75]); the 1-week Delay condition improved from 0.19 (*SE* = 0.03, 95% CI = [0.14, 0.24]) to 0.52 (*SE* = 0.04, 95% CI = [0.43, 0.61]); the 2-week Delay condition improved from 0.15 (*SE* = 0.02, 95% CI = [0.10, 0.20]) to 0.65 (*SE* = 0.04, 95% CI = [0.56, 0.73]). The interaction between Item Type and Delay, which might have indicated that the size of the retrieval-induced facilitation effect varied as a function of Delay, was not significant, *F*(2, 96) = 1.93, *η*_*p*_^2^ = 0.04, *p* = 0.15. Although this interaction did not meet the level of statistical significance, some numerical trends are worth noting. As shown in Fig. 3, participants in the 2-week Delay condition were the only group to demonstrate significant evidence of retrieval-induced facilitation, *t*(32) = 2.42, *p* = 0.02, 95% CI_difference_ = [0.01, 0.11], *d* = 0.42, BF_10_ = 2.30. This pattern was not shown for the 1-day Delay condition, *t*(34) = -0.78, *p* = 0.44, 95% CI_difference_ = [− 0.07, 0.03], *d* = − 0.13, BF_01_ = 4.16, or the 1-week Delay condition, *t*(30) = 0.12, *p* = 0.91, 95% CI_difference_ = [− 0.07, 0.08], *d* = 0.02, BF_01_ = 5.21.

The interaction between Item Type and Test was not significant. In other words, the difference in recall between Rp− and Nrp items did not vary as a function of Test: *F*(1, 96) = 0.69, *p* = 0.41, *η*_*p*_^2^ = 0.01. Finally, there was no evidence of an interaction between Item Type, Test, and Delay: *F*(2, 96) = 0.2, *p* = 0.82, *η*_*p*_^2^ = 0.00. There was no evidence that the size of the retrieval-induced facilitation effect (or the difference between Rp− and Nrp items) changed from Test 1 to Test 2 depending on Delay condition.

#### Experiment 2b

A 2 (Item Type: Rp-, Nrp; within-subjects) × 2 (Test: Test 1, Test 2; within-subjects) × 3 (Delay: 1 day, 1 week, 2 weeks; between-subjects) ANOVA was performed. Not surprisingly, performance was better on Test 2 (*M* = 0.58, SE = 0.02) than on Test 1 (*M* = 0.28, *SE* = 0.02): *F*(1, 96) = 250.53,* p* < 0.001, *η*_*p*_^2^ = 0.72, 95% CI_difference_ = [0.27, 0.34]. There was also a main effect of Delay, *F*(2, 96) = 3.66, *p* = 0.03, *η*_*p*_^2^ = 0.07. Participants in the 1-day Delay condition (*M* = 0.49, *SE* = 0.03) performed better than participants in the 2-week (*M* = 0.42, *SE* = 0.03) and 1-week (*M* = 0.38, *SE* = 0.03) Delay conditions. A main effect of Item Type was also observed, *F*(1, 96) = 18.72, *p* < 0.001, *η*_*p*_^2^ = 0.16. Rp- items (*M* = 0.47, *SE* = 0.02) were recalled significantly better than Nrp items (*M* = 0.39, *SE* = 0.02; 95% CI_difference_ = [0.04, 0.12]). Importantly, this pattern provides evidence of retrieval-induced facilitation.

**Fig. 4:**
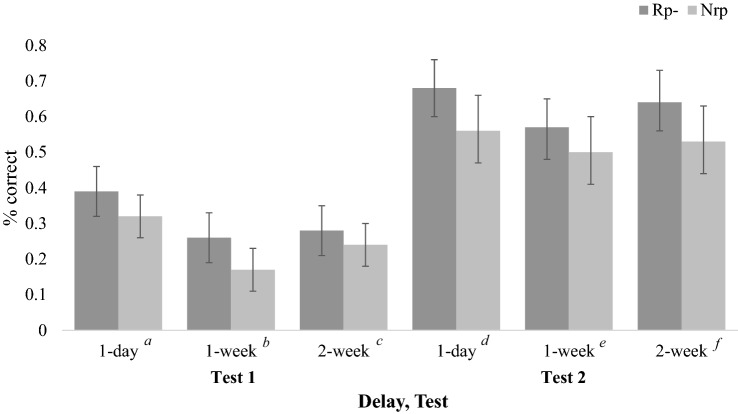
Experiment 2b paired samples t-tests showing performance by delay and test. Error bars show standard errors. ^a^*t*(34) = 2.49, *p* = 0.02, 95% CI_difference_ = [0.01, 0.13], BF_10_ = 2.62. ^b^*t*(30) = 2.67, *p* = 0.01, 95% CI_difference_ = [0.02, .16], BF_10_ = 3.78. ^c^*t*(32) = 1.01, *p* = 0.32, 95% CI_difference_ = [− 0.04, 0.11], BF_01_ = 3.36. ^d^*t*(34) = 3.11, *p* = 0.00, 95% CI_difference_ = [0.04, 0.19], BF_10_ = 9.66. ^e^*t*(30) = 1.41, *p* = 0.17, 95% CI_difference_ = [− 0.03, 0.15], BF_01_ = 2.16. ^f^*t*(32) = 1.92, *p* = 0.06, 95% CI_difference_ = [− 0.01, 0.23], BF_01_ = 1.05.

No interactions met the level of statistical significance. The difference between Rp− and Nrp did not differ between the three Delay conditions: *F*(2, 96) = 0.10, *p* = 0.90, *η*_*p*_^2^ = 0.00. As shown in Fig. 4, participants in the 1-day Delay condition exhibited significant retrieval-induced facilitation, *t*(34) = 4.20, *p* < 0.001, 95% CI_difference_ = [0.05, 0.14], *d* = 0.71, BF_10_ = 146.07, as did participants in the 1-week Delay condition, *t*(30) = 2.49, *p* = 0.02, 95% CI_difference_ = [0.01, 0.14], *d* = 0.45, BF_10_ = 2.60. Although the effect did not reach the level of statistical significance, participants in the 2-week Delay condition trended toward retrieval-induced facilitation, *t*(32) = 1.76, *p* = 0.09, 95% CI_difference_ = [− 0.01, 0.16], *d* = 0.31 (95% CI = [− 0.05, 0.65]), BF_01_ = 1.35.

The increase in recall performance from Test 1 to Test 2 did not vary based on Delay: *F*(2, 96) = 1.04, *p* = 0.36, *η*_*p*_^2^ = 0.02. Additionally, neither Rp− nor Nrp items showed a greater increase from Test 1 to Test 2: *F*(1, 96) = 0.97, *p* = 0.33, *η*_*p*_^2^ = 0.01. Finally, no three-way interaction was observed. The size of the retrieval-induced facilitation effect did not change from Test 1 to Test 2 differently depending on Delay: *F*(2, 96) = 1.19, *p* = 0.31, *η*_*p*_^2^ = 0.02.

### Discussion

The results of Experiments 2a and 2b provide some evidence of retrieval-induced facilitation, replicating the general pattern observed by Chan et al. (2006) and Chan (2009). The results were most compelling in Experiment 2b. There was no significant evidence of the retrieval-induced facilitation effect becoming larger on Test 2 compared to Test 1. That said, it is noteworthy that the effect was not eliminated by feedback on Test 1. Re-exposure to Rp− and Nrp items might have eliminated the effect of retrieval-induced facilitation in the same way that re-exposure to Rp− items and Nrp items has been shown to eliminate retrieval-induced forgetting (Storm et al., 2008, 2012; Storm & Soares, 2022). The current results suggest that whereas the costs of retrieval practice can be eliminated by relearning, the benefits of retrieval practice may not be—a pattern of results that makes sense given the theoretical distinction between the cause of retrieval-induced forgetting and the cause of retrieval-induced facilitation.

Finally, it is unclear why evidence of retrieval-induced facilitation was observed in Experiments 2a and 2b but not in Experiments 1a and 1b. One possibility is that the effect is small and variable, and by chance it was significant in one experiment but not the other. It is worth noting that although statistically significant, the magnitude of the retrieval-induced facilitation effects that we observed were substantially smaller than the 9 and 11% effects reported by Chan (2009). Another possibility is that there were subtle differences in the experimental procedure or sample. Experiments 2a and 2b were run entirely online during the COVID-19 pandemic, for example, whereas Experiments 1a and 1b were run in the laboratory. Given the cross-experiment comparison, it is impossible to know for sure, but this difference could be something for future research to consider.

### Meta-analysis

Taken together, the results of the current experiments suggest that retrieval-induced facilitation may be a real effect, just one that is somewhat smaller than previously observed. Chan’s (2009) two experiments included a combined total of 48 participants in the critical condition (high integration/long delay). In contrast, the current two experiments included a combined total of 164 participants in the critical condition. Moreover, all 164 participated in two experiments designed to measure retrieval-induced facilitation, yielding 328 data points. As a result, although our individual experiments were somewhat limited in power, the current study when combined across experiments had far more power to estimate the true effect size of retrieval-induced facilitation than the study reported by Chan. Indeed, the fact that Chan’s study was so underpowered was a large part of the motivation for conducting the current work.

Given the broad theoretical implications for understanding the consequences of retrieval and the potential everyday applications to contexts like the classroom, it is important to estimate the magnitude of the retrieval-induced facilitation effect more accurately. A meta-analysis was used for this purpose, including the combined data reported by Chan (2009) and the two sets of experiments reported here. Specifically, the spreadsheet provided by Neyeloff et al. (2012) was used to perform the meta-analysis calculations and construct the accompanying forest plot. Included as separate “experiments” were each of the delay conditions for both tasks from both current experiments: 4 “experiments” from Experiments 1a and 1b (Experiment 1a, 1 day and 2 weeks; Experiment 1b, 1 day and 2 weeks) and 6 “experiments” from Experiments 2a and 2b (Experiment 2a, 1 day, 1 week, and 2 weeks; Experiment 2b, 1 day, 1 week, and 2 weeks). Note that only the results from Test 1 in Experiments 2a and 2b were used, not Test 2. Chan’s (2009) results from the high-integration/24-h delay conditions were also included. Thus, the meta-analysis included a total of 12 experiments (*k* = 12; *df* = 11).

Results from the meta-analysis are shown in Table 1. Sample sizes ranged from 24 to 35 (*M* = 32); effect sizes ranged from − 0.04 to 0.11 (*M* = 0.04); standard error ranged from 0.03 to 0.07 (*M* = 0.04). The measure of the effect size was the difference between performance on Rp− items and Nrp items (so, in the two instances showing a retrieval-induced forgetting rather than a retrieval-induced facilitation, the score is negative).

**Table 1:**
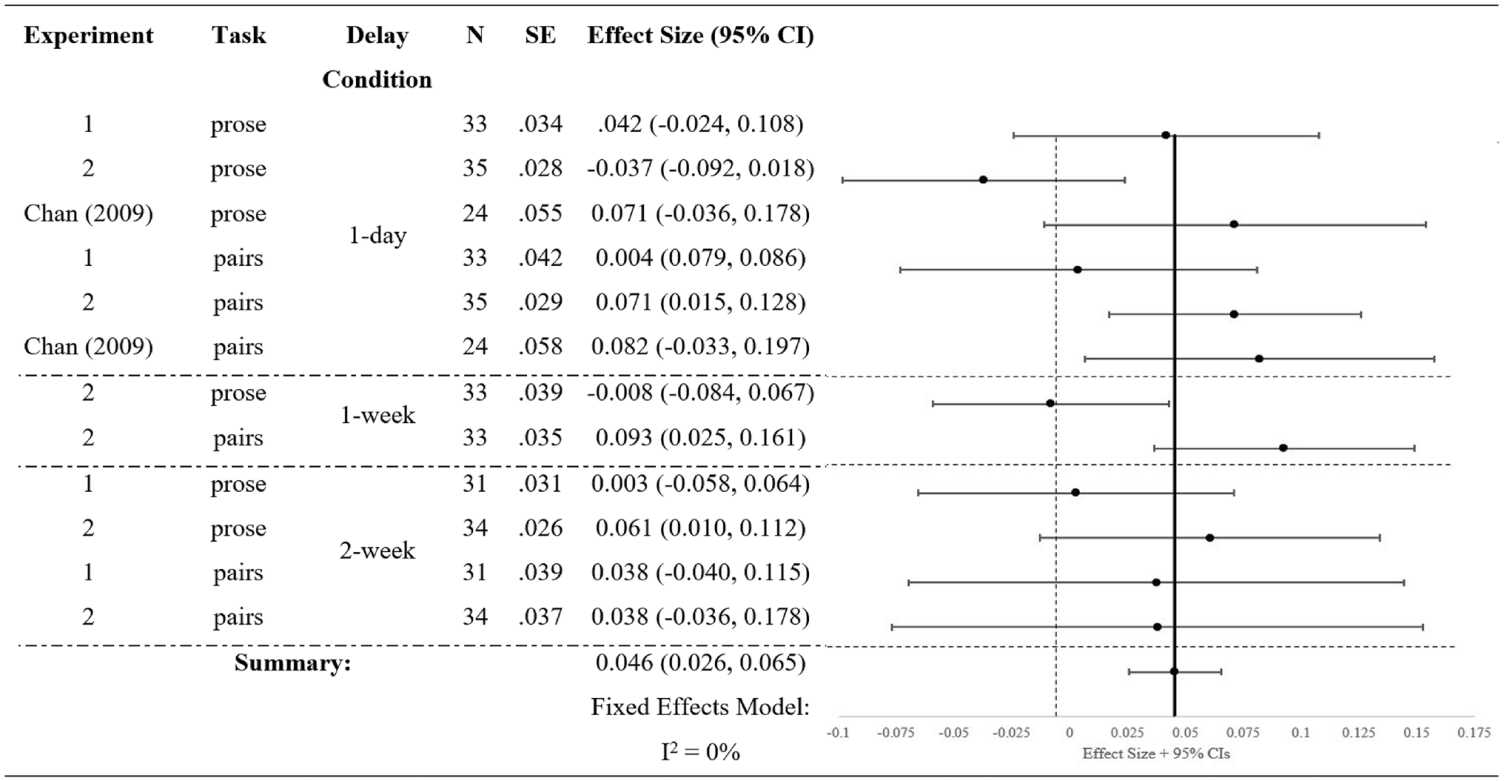
Meta-analysis results arranged by delay

The measure of heterogeneity in samples, *Q*, was calculated to be 8.44, which was below the critical value for the size of the sample (19.68), so there was no evidence to support true between-studies variability. To quantify more specifically any heterogeneity, *I*^2^ was calculated to be − 30.20. Common practice dictates that this negative number can be interpreted to indicate that 0% of the variability in effect sizes that was observed in the meta-analysis was due to true heterogeneity in the included samples (Higgins et al., 2003). Thus, a fixed-effects model was used rather than a random-effects model. Taking into account the variance and sample size of each experiment, the weighted effect size of retrieval-induced facilitation was 0.05 (SE = 0.01, 95% CI = [0.03, 0.07]). The same calculations were performed with Chan’s experiments removed (i.e., only including the results from our experiments), and this analysis showed a slightly smaller but still significant average effect size of 0.04 (95% CI = [0.02, 0.06]).

Although the heterogeneity statistic did not suggest differences between delays or tasks, the effect sizes are reported for illustrative purposes. The effect size was 0.05 (95% CI = [0.02, 0.08]) in the 1-day delay condition, 0.05 (95% CI = [0.00, 0.11]) in the 1-week delay condition, and 0.04 (95% CI = [0.01, 0.07]) in the 2-week delay condition. These results suggest that retrieval-induced facilitation’s effect size did not vary as a function of the delay between retrieval practice and test. It remained significant at each time point, although power is limited due to the small number of “experiments” at each Delay.

The meta-analysis was also broken down by task. When considering the experiments using prose materials (i.e., Experiment 1a, Experiment 2a, and Chan’s Experiment 1), the estimated effect size was 0.04 (95% CI = [0.01, 0.06]). For the experiments using object–location pairs (i.e., Experiment 1b, Experiment 2b, and Chan’s Experiment 2), the estimated effect size was 0.06 (95% CI = [0.03, 0.09]).

## General discussion

Most research on the consequences of retrieval practice has focused on two major findings: (1) that retrieval tends to make retrieved items more recallable in the future than they would have been otherwise (i.e., the testing effect; Roediger & Karpicke, 2006a), and (2) that retrieval tends to make other, non-retrieved items less recallable in the future than they would have been otherwise (i.e., retrieval-induced forgetting; Anderson, 2003). Although these two consequences are quite robust (Murayama et al., 2014; Roediger et al., 2011; Rowland, 2014; Storm et al., 2015), there are conditions under which a different pattern of results emerges. Specifically, when to-be-learned materials are well integrated, and when the final test is delayed by 24 h, non-retrieved items have been shown to become more recallable in the future than they would have been otherwise (i.e., retrieval-induced facilitation; Chan, 2009; Chan et al., 2006).

The goal of the current study was to replicate and extend evidence of the retrieval-induced facilitation effect, providing the literature with a more precise estimate of its true effect size. Whereas the mitigating effects of integration and delay on retrieval-induced forgetting are well established, evidence pertaining to their combined effect leading to retrieval-induced facilitation is not. Indeed, only a small handful of studies have been specifically designed to study retrieval-induced facilitation. Although the current results provide some evidence of retrieval-induced facilitation, the size of the effect was substantially smaller than that which has been previously observed. Specifically, collapsed across all four current experiments, the estimated effect size was 4% (95% CI = [2%, 6%]), compared to the 7–11% effect sizes observed by Chan (2009) and Chan et al. (2006). The reduced effect size should not be surprising, however, given that replication studies overall tend to produce effect sizes smaller than those initially reported (Patil et al., 2016). Interestingly, there was neither evidence of the effect varying as a function of final delay (1 day vs. 1 week vs. 2 weeks) nor was there evidence of the effect becoming larger on a second test following feedback (Experiments 2a and 2b).

The finding of significant retrieval-induced facilitation effects after 1- and 2-week delays (at least when looking at the results of the meta-analysis) is important. It fits well with the idea that retrieval-induced facilitation is caused by the activation of non-practiced items during retrieval practice, thereby giving non-practiced items a kind of covert retrieval practice that enhances their subsequent accessibility. Just as testing effects have been shown to remain significant across a long delay (Roediger & Karpicke, 2006b), so too, it appears, do the effects of retrieval-induced facilitation. This finding provides an interesting juxtaposition to retrieval-induced forgetting, which has generally been observed to become diminished over time.

In the first study to show evidence of retrieval-induced facilitation, Chan et al. (2006) employed prose passages (indeed, they were the same passages as those used by Chan (2009) and the current Experiments 1a and 2a) and found 9% and 7% effects of retrieval-induced facilitation in their Experiments 1 and 2, respectively. In their Experiment 3, Chan et al. manipulated the strategy employed by participants during retrieval practice (broad vs. narrow). Specifically, participants were asked to either minimize or maximize the breadth of their retrieval efforts (i.e., the extent to which they attempted to think of related facts for each question). Participants who used a broad strategy demonstrated a 10% retrieval-induced facilitation effect, whereas participants who used a narrow strategy demonstrated a 0% retrieval-induced facilitation effect. This finding suggests that facilitation effects may be larger when participants are led to consciously think about related information during retrieval. Interestingly, it does not appear that Chan gave participants the instruction to engage in broad retrieval practice in his 2009 experiments (and the current study followed suit), choosing to measure the consequences of retrieval practice when participants are not given any specific instructions at the time of retrieval practice. It is possible that a larger and more consistent effect of retrieval-induced facilitation would have been observed if, as part of the current study, participants were instructed to undertake broad retrieval practice. Then, they may have been more likely to activate related information from the passage.

Another potential explanation for the relatively smaller effect sizes observed in our experiments compared to those by Chan et al. may be related to differences in the samples employed and how participants engaged in the retrieval-practice tasks. If participants in the studies reported by Chan et al. were more likely to naturally engage in the kind of broad retrieval strategies that promote retrieval-induced facilitation than the participants in our experiments, for example, then such a difference could have contributed to the discrepant effect sizes observed across the two studies.

It is worth noting that several studies have used other paradigms to observe evidence of retrieval-induced facilitation. In the work by Bäuml and Samenieh (2010, 2012), for example, retrieval practice was shown to enhance memory for items that suffered from either directed forgetting or context-dependent forgetting, presumably because retrieval reactivated certain contextual cues and therefore facilitated access to the individual items within that context. Similar effects have been observed in studies using categorical or contextually bound materials and free recall tests (e.g., Rowland & DeLosh, 2014). For example, retrieval practice can make participants more likely to think of practiced categories than non-practiced categories on a free recall test, leading to a retrieval-induced facilitation effect that is caused not by the retrieval-induced activation of individual items, but by the increased accessibility of practiced categories at final test. The current results (combined with those reported by Chan and colleagues) are important in that they suggest that retrieval-induced facilitation can also result from the activation of individual items during retrieval practice.

From an applied standpoint, the current results suggest that retrieval does not always cause the forgetting of non-retrieved information in memory. Instead, under the right conditions, retrieval has the potential to enhance the subsequent remembering of non-retrieved information (for related evidence in a classroom setting, see Cranney et al., 2009). The information studied by participants in these experiments reflected educational materials, and the long delays between retrieval practice and test (ranging from one day to two weeks) also reflected a typical student’s studying habits, making these findings particularly relevant to the classroom. With the testing effect supporting the recall of practiced information, and retrieval-induced facilitation supporting the recall of related and unpracticed information, teachers and students would be well served to take advantage of retrieval practice (for example, through practice tests) to support learning.

The knowledge that retrieval-induced facilitation is a possible outcome of retrieval practice may help to allay concerns about the potential unintended effects of retrieval-induced forgetting. Indeed, it is possible that under typical educational settings—where materials tend to be cohesive and well integrated, and where learning is assessed following delays longer than a few minutes—retrieval-induced facilitation may occur far more often than retrieval-induced forgetting. To extend this work further, future research should explore (1) the extent to which retrieval-induced facilitation is observed in actual everyday learning environments, and (2) whether there are conditions that can be implemented during learning and retrieval practice to magnify the effects of retrieval-induced facilitation that are observed.

## Data Availability

The data can be found on the Open Science Framework at https://osf.io/d8gsh/.

## References

[CR1] Anderson JR (1983). The architecture of cognition.

[CR2] Anderson MC (2003). Rethinking interference theory: Executive control and the mechanisms of forgetting. Journal of Memory and Language.

[CR3] Anderson MC, Bjork RA, Bjork EL (1994). Remembering can cause forgetting: Retrieval dynamics in long-term memory. Journal of Experimental Psychology: Learning, Memory, and Cognition.

[CR4] Anderson MC, Green C, McCulloch KC (2000). Similarity and inhibition in long-term memory: Evidence for a two-factor model. Journal of Experimental Psychology: Learning, Memory, and Cognition.

[CR5] Anderson MC, McCulloch KC (1999). Integration as a general boundary condition on retrieval-induced forgetting. Journal of Experimental Psychology: Learning, Memory, and Cognition.

[CR6] Bäuml K-HT, Samenieh A (2010). The two faces of memory retrieval. Psychological Science.

[CR7] Bäuml K-HT, Samenieh A (2012). Selective memory retrieval can impair and improve retrieval of other memories. Journal of Experimental Psychology: Learning, Memory, and Cognition.

[CR8] Bjork RA, Bjork EL (1992). A new theory of disuse and an old theory of stimulus fluctuation. From learning processes to cognitive processes: Essays in honor of William K. Estes.

[CR9] Butler AC, Roediger HL (2007). Testing improves long-term retention in a simulated classroom setting. European Journal of Cognitive Psychology.

[CR10] Carroll M, Campbell-Ratcliffe J, Murnane H, Perfect T (2007). Retrieval-induced forgetting in educational contexts: Monitoring, expertise, text integration, and test format. European Journal of Cognitive Psychology.

[CR11] Chan JC (2009). When does retrieval induce forgetting and when does it induce facilitation? Implications for retrieval inhibition, testing effect, and text processing. Journal of Memory and Language.

[CR12] Chan JC, McDermott KB, Roediger HL (2006). Retrieval-induced facilitation: Initially nontested material can benefit from prior testing of related material. Journal of Experimental Psychology: General.

[CR13] Collins AM, Loftus EF (1975). A spreading-activation theory of semantic processing. Psychological Review.

[CR14] Congleton A, Rajaram S (2012). The origin of the interaction between learning method and Delay in the testing effect: The roles of processing and conceptual retrieval organization. Memory & Cognition.

[CR15] Cranney J, Ahn M, McKinnon R, Morris S, Watts K (2009). The testing effect, collaborative learning, and retrieval-induced facilitation in a classroom setting. European Journal of Cognitive Psychology.

[CR16] García-Bajos E, Migueles M, Anderson MC (2009). Script knowledge modulates retrieval-induced forgetting for eyewitness events. Memory.

[CR17] Goodmon LB, Anderson MC (2011). Semantic integration as a boundary condition on inhibitory processes in episodic retrieval. Journal of Experimental Psychology: Learning, Memory, and Cognition.

[CR18] Higgins JP, Thompson SG, Deeks JJ, Altman DG (2003). Measuring inconsistency in meta-analyses. British Medical Journal.

[CR19] Jonker TR, Seli P, MacLeod CM (2013). Putting retrieval-induced forgetting in context: An inhibition-free, context-based account. Psychological Review.

[CR20] MacLeod MD, Macrae CN (2001). Gone but not forgotten: The transient nature of retrieval-induced forgetting. Psychological Science.

[CR21] Migueles M, García-Bajos E (2007). Selective retrieval and induced forgetting in eyewitness memory. Applied Cognitive Psychology.

[CR22] Murayama K, Miyatsu T, Buchli D, Storm BC (2014). Forgetting as a consequence of retrieval: A meta-analytic review of retrieval-induced forgetting. Psychological Bulletin.

[CR23] Neyeloff JL, Fuchs SC, Moreira LB (2012). Meta-analyses and Forest plots using a Microsoft Excel spreadsheet: Step-by-step guide focusing on descriptive data analysis. BMC Research Notes.

[CR24] Patil P, Peng RD, Leek JT (2016). What should researchers expect when they replicate studies? A statistical view of replicability in psychological science. Perspectives on Psychological Science.

[CR25] Raaijmakers JG, Jakab E (2013). Rethinking inhibition theory: On the problematic status of the inhibition theory for forgetting. Journal of Memory and Language.

[CR26] Roediger HL, Karpicke JD (2006). The power of testing memory: Basic research and implications for educational practice. Perspectives on Psychological Science.

[CR27] Roediger HL, Karpicke JD (2006). Test-enhanced learning: Taking memory tests improves long-term retention. Psychological Science.

[CR28] Roediger HL, Putnam AL, Smith MA (2011). Ten benefits of testing and their applications to educational practice. The Psychology of Learning and Motivation: Cognition in Education.

[CR29] Rowland CA (2014). The effect of testing versus restudy on retention: A meta-analytic review of the testing effect. Psychological Bulletin.

[CR30] Saunders J, Fernandes M, Kosnes L (2009). Retrieval-induced forgetting and mental imagery. Memory & Cognition.

[CR31] Saunders J, MacLeod MD (2002). New evidence on the suggestibility of memory: The role of retrieval-induced forgetting in misinformation effects. Journal of Experimental Psychology: Applied.

[CR32] Storm BC, Angello G, Buchli DR, Koppel RH, Little JL, Nestojko JF (2015). A review of retrieval-induced forgetting in the contexts of learning, eye-witness memory, social cognition, autobiographical memory, and creative cognition. The Psychology of Learning and Motivation.

[CR33] Storm BC, Bjork EL, Bjork RA (2008). Accelerated relearning after retrieval-induced forgetting: The benefit of being forgotten. Journal of Experimental Psychology: Learning, Memory, and Cognition.

[CR34] Storm BC, Bjork EL, Bjork RA (2012). On the durability of retrieval-induced forgetting. Journal of Cognitive Psychology.

[CR35] Storm BC, Levy BJ (2012). A progress report on the inhibitory account of retrieval-induced forgetting. Memory & Cognition.

[CR36] Storm BC, Soares JS (2022). Relearning can eliminate the effect of retrieval-induced forgetting. Psychological Research.

[CR37] Verde MF (2013). Retrieval-induced forgetting in recall: Competitor interference revisited. Journal of Experimental Psychology: Learning, Memory, and Cognition.

[CR38] Wheeler M, Ewers M, Buonanno J (2003). Different rates of forgetting following study versus test trials. Memory.

